# Acute unilateral DBS of the basolateral amygdala does not affect motricity, anxiety-like behavior, or fear memory consolidation in rats

**DOI:** 10.1007/s00221-026-07408-5

**Published:** 2026-10-12

**Authors:** Douglas Affonso Formolo, Nicholas Roberto Drabowski, Hiago Murilo Mello, Alexandre Ademar Hoeller, Cristiane Ribeiro de Carvalho, Alexandre Trofino Neto, Roger Walz

**Affiliations:** 1 Department of Counselling and Psychology, Hong Kong Shue Yan University, Hong Kong, China; 2 Graduation Program of Neuroscience, Center of Biological Sciences, Federal University of Santa Catarina (UFSC), Florianópolis, Santa Catarina 88040-970 Brazil; 3 Center of Applied Neuroscience (CeNAp), University Hospital, UFSC, Florianópolis, Santa Catarina Brazil; 4 Graduate Program in Automation and Systems Engineering, Technological Center, UFSC, Florianópolis, Santa Catarina Brazil; 5 Department of Internal Medicine, Center of Health Sciences, University Hospital, Federal University of Santa Catarina, 3rd floor, Florianópolis, Santa Catarina 88040-970 Brazil; 6 Graduation Program in Medical Sciences, Center of Health Sciences, University Hospital, Federal University of Santa Catarina, Florianópolis, Santa Catarina Brazil; 7 Department of Systems and Automation, Technological Center, UFSC, Florianópolis, Santa Catarina Brazil; 8 Department of Pathology, Center of Health Sciences, Federal University of Santa Catarina, Florianópolis, Santa Catarina Brazil

**Keywords:** Deep brain stimulation (DBS), Amygdala, Basolateral amygdala, Anxiety, Fear memory

## Abstract

Deep brain stimulation (DBS) is an alternative to ablative functional neurosurgeries. The basolateral amygdala (BLA) is a potential DBS target for treating psychiatric disorders. However, understanding the associated behavioral and cognitive effects is necessary for safety and tolerability concerns. Therefore, we investigated potential acute effects of unilateral BLA DBS. Adult male Wistar rats were implanted with monopolar electrodes in the right BLA. In Experiment 1, different groups of animals were stimulated for 10 min with either high-frequency (HFS, 130 Hz) or low-frequency (LFS, 20 Hz) stimulation using different stimulation intensities (0 [Control], 10, 50, or 100 µA). Immediately after stimulation, animals were tested in the elevated plus-maze (EPM) and subsequently in the open field test (OFT). In Experiment 2, two groups of animals were subjected to a fear-conditioning protocol and stimulated with HFS or LFS (50 µA) immediately after conditioning. Animals were then tested for short- (2 h) and long-term (24 h) fear memory consolidation. BLA DBS showed no significant effects on: (i) anxiety-like behavior expression for HFS (*p* = 0.46) or LFS (*p* = 0.37); (ii) locomotor activity (HFS, *p* = 0.12; LFS, *p* = 0.60), in terms of distance traveled in the OFT, or (iii) fear memory consolidation (HFS, *p* = 0.31; LFS, *p* = 0.68). Under the stimulation parameters and experimental conditions tested, acute unilateral BLA DBS did not significantly affect spontaneous locomotor activity, anxiety-like behavior, or fear memory consolidation in rats. These results provide information relevant to the tolerability and safety of amygdala DBS.

## Introduction

Electroceutics is a growing field in which electrical impulses are used therapeutically to modulate neural circuits (Famm et al. 2013). Deep brain stimulation (DBS) is one such technique, consisting of the direct application of flexible, reversible electrical impulses through surgically implanted electrodes in deep brain structures (Benabid et al. 1987; Miocinovic et al. 2013). The interaction between brain tissue, electrode geometry, and stimulation parameters results in a complex modulation of local and distant networks, leading to both acute and long-term therapeutic effects (Brocker and Grill 2013; Hamani and Temel 2012; Kuncel and Grill 2004; Ranck 1975). DBS has been successfully used to treat movement disorders, including advanced Parkinson’s disease, essential tremor, and dystonia, via high-frequency stimulation (HFS) of the subthalamic nucleus (STN) and globus pallidus pars interna (GPi) (Meoni et al. 2017; Odekerken et al. 2013). It is also approved for the treatment of obsessive-compulsive disorder (Alonso et al. 2015) and refractory epilepsy (Wu et al. 2021), and it is under investigation for different treatment-resistant disorders, such as depression, addiction, stress-related disorders, and obesity (Formolo et al. 2019; Luigjes et al. 2013).

Due to its reversibility, DBS has virtually replaced ablative functional neurosurgeries (Hariz and Hariz 2013). Nonetheless, a comprehensive understanding of the mechanisms of action of DBS remains elusive. Whereas GPi DBS, for instance, results in instant motor improvements in the tremor and rigidity of patients with Parkinson’s disease (Boraud et al. 1996; Iacono et al. 1995), the stimulation of the same structure and with similar parameters takes several weeks or months to achieve motor improvements in patients with dystonia (Coubes et al. 2000). The stimulation outcome is therefore highly specific to the targeted structure and underlying pathology, requiring equally specific stimulation parameters. DBS’s promising results led to a rapid spread in its clinical use, and additional preclinical research will be useful for improving clinical practice (Hamani and Temel 2012).

In this context, the experimental investigation of DBS has helped explain the interplay among stimulation protocol, target structure, and behavioral outcomes. Hamani and colleagues, for instance, demonstrated that depression-like behavior as measured by the forced swim test is sensitive to DBS of the ventromedial prefrontal cortex (vmPFC), experimentally reproducing the ongoing clinical investigations of subcallosal cingulate cortex DBS as a treatment for depression (Hamani et al. 2010). Nevertheless, behavioral changes depended on the stimulation protocol, with the best behavioral response obtained with HFS (130 Hz) and the least with low-frequency stimulation (LFS, 20 Hz) (Hamani et al. 2010). When addressing motor-related outcomes through DBS of motor areas (e.g., STN) in animal models of Parkinson’s disease, it is not uncommon to observe opposing effects between HFS and LFS, with HFS improving motor symptoms and LFS worsening them (Zhang et al. 2024). Curiously, while DBS of distinct brain regions may lead to comparable behavioral results (Hamani et al. 2014), the stimulation of different nuclei within the same brain region can lead to different outcomes (Hamani et al. 2010). While vmPFC or nucleus accumbens high-frequency DBS can have comparable antidepressant-like effects (Hamani et al. 2014), within the vmPFC the effects arise from selective stimulation of the prelimbic subregion but not the infralimbic (Hamani et al. 2010). Finally, in the basolateral nucleus of the amygdala (BLA), high-frequency DBS administered after conditioning reduced the retrieval of auditory but not contextual fear memory, suggesting a modality-specific behavioral effect within a single target (Sui et al. 2014). Together, these studies illustrate that DBS outcomes are highly sensitive to the combined influence of target location and stimulation parameters, which underscores the importance of systematically characterizing the behavioral profile of each candidate DBS target before clinical translation.

The amygdala is the cornerstone of the survival neurocircuit, a system intertwined for detecting and responding to threats (de Carvalho et al. 2021; Fox and Shackman 2024; Izquierdo et al. 1993; Leal et al. 2018; LeDoux 2012; Walz et al. 2000). It is composed of multiple nuclei, including the BLA, which is extensively involved in evaluating stimulus significance, and the central nucleus of the amygdala (CeA), the output region connected to structures involved in autonomic responses (Janak and Tye 2015). In humans, the amygdala’s dysfunctional hyperactivity has been associated with different pathologies, including temporal lobe epilepsy (TLE) (Gotman and Levtova 1996; Leal et al. 2018), post-traumatic stress disorder (PTSD) (Ross et al. 2017), and autism spectrum disorder (ASD) (Hennessey et al. 2018). Likewise, BLA DBS for such conditions has been reported to successfully reduce disease symptoms in case reports and small-scale population studies (Boon et al. 2007; Langevin et al. 2016a, b; Sturm et al. 2013; Vonck et al. 2002). Moreover, BLA DBS is currently under investigation in a clinical trial for treatment-resistant PTSD in war combatants (Koek et al. 2014; Langevin et al. 2016a, b). Preliminary results of two subjects who have completed the four-year trial indicated improvements of up to 55% in symptom severity and reengagement in social and professional activities, suggesting amygdala DBS as an effective strategy for severe, treatment-resistant PTSD (Koek et al. 2024).

As DBS is classified as a high-risk device under FDA and international regulatory frameworks (Pena et al. 2007), the development of new clinical applications demands rigorous preclinical safety evidence (Cogan et al. 2016; Shepherd et al. 2018). A relevant safety benchmark in this context is the widely accepted Shannon–McCreery limit of 30 µC/cm² per phase as the upper boundary of the safe charge-density range for macrostimulation of neural tissue (Cogan et al. 2016). Despite their relevance, such safety guidelines do not prevent stimulation-related side effects. Cognitive decline and depressed mood have been observed after long-term DBS for both Parkinson’s disease (Volkmann et al. 2009) and refractory epilepsy (Fisher et al. 2010). However, they usually come with a lower burden than the otherwise treatment-resistant disease. Nonetheless, severe acute side effects, such as panic attacks that can arise from DBS in the nucleus accumbens or the anterior limb of the internal capsule (Okun et al. 2007; Shapira et al. 2006), can render the treatment ineffective from the outset. Such acute side effects and stimulation tolerability are tested in clinical settings using brief stimulation during surgical electrode placement (Schlaepfer et al. 2014) and during acute monopolar survey before chronic stimulation starts (Greenberg et al. 2010). Stimulation is performed over a few minutes and can result in both positive and negative cognitive and/or mood-altering effects (Greenberg et al. 2010; Schlaepfer et al. 2014). BLA DBS is particularly concerning for acute side effects, as the amygdala sits at the center of the survival neurocircuit. Indeed, in an ongoing PTSD trial, changing the intensity of the BLA stimulation shifted the subjective emotional response from overall happiness to strong negative emotions resembling those felt during PTSD flashbacks (Lai et al. 2020). Despite such anecdotal evidence from case studies, preclinical evidence for acute BLA effects is still scant.

On the other extreme, clinical trials must also account for the placebo effect. As recently meta-analyzed, DBS for treatment-resistant OCD is associated with significant non-intervention-related effects in sham-stimulated patients (Schruers et al. 2019), underscoring the relevance of preclinical investigations for both safety evidence and the control of expectancy-driven results.

The BLA is involved in the expression of anxiety-like behavior in rodents (Pellow et al. 1985; Walf and Frye 2007). Notably, we previously demonstrated that the same neurochemical changes in specific signal transduction in the BLA observed in rodents immediately after the expression of anxiety-like behavior have a clinical correlate with anxiety symptoms in humans (de Carvalho et al. 2021). Moreover, modulation of fear memory consolidation and its expression in rodents also requires amygdala-dependent mechanisms (de Carvalho et al. 2021; Duvarci and Pare 2014; Inman et al. 2018; Walz et al. 2000). Therefore, we investigated possible acute behavioral and cognitive effects induced by different stimulation parameters of unilateral BLA DBS, including motricity, anxiety-like behavior, and fear memory consolidation. Given the commonly observed opposing effects of HFS (local inhibition-like effect) and LFS (local excitation-like effect) and the functional relevance of the BLA in modulating survival behavior, we hypothesized that low-frequency BLA DBS would elicit acute anxiety-like behaviors and enhance fear memory consolidation, whereas HFS would lead to opposite outcomes. Contrary to our expectations, neither high- nor low-frequency BLA DBS affected the measured behaviors at the various stimulation intensities tested.

## Materials and methods

### Experimental design

**Fig. 1:**
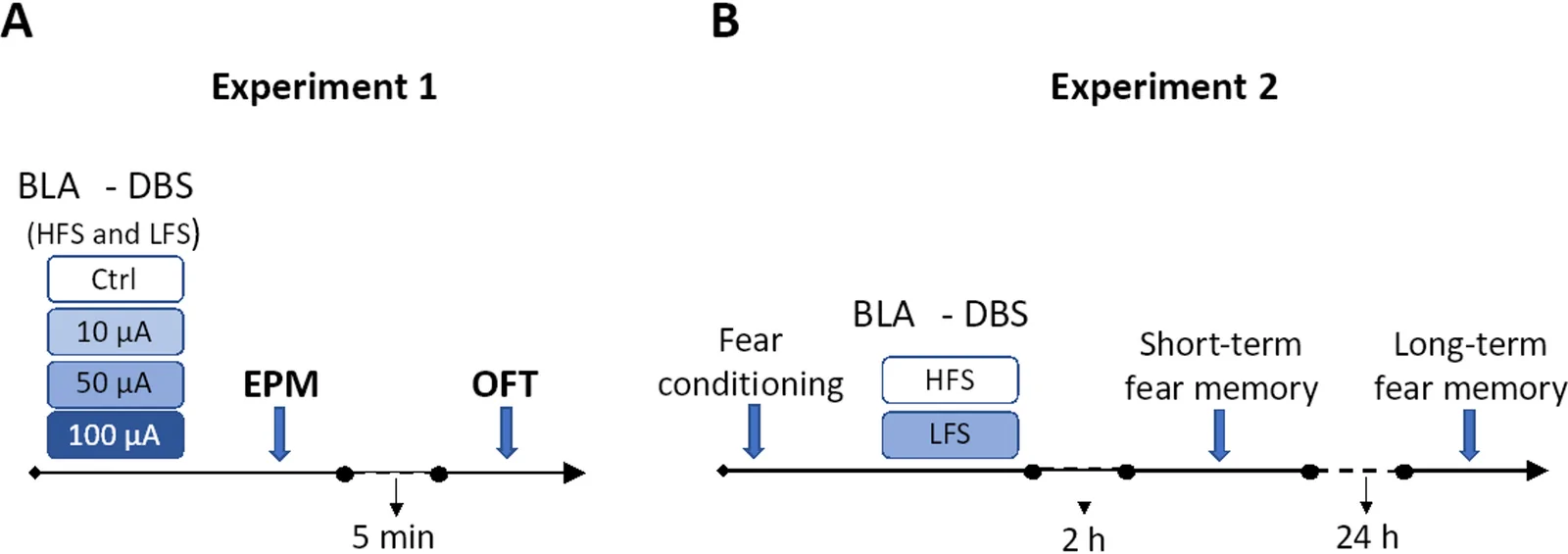
Experimental design. In Experiment 1, independent samples were grouped based on stimulation intensity (0, [Ctrol], 10, 50, or 100 µA) and stimulation frequency (HFS, 130 Hz; LFS, 20 Hz). Animals were immediately submitted to the elevated plus-maze (EPM) and open-field (OFT) tests after the 10-minute stimulation period, with a 5-min interval between tests. In Experiment 2, independent samples were stimulated with HFS or LFS immediately after the fear conditioning protocol. Two and 24 h after fear conditioning, animals were assessed for short- and long-term fear memory

Stimulation and behavioral tests were conducted between 1 and 6 p.m., after the animals had habituated for 1 h in the experimental room. In Experiment 1, we tested the potential anxiogenic effects of acute HFS (130 Hz) or LFS (20 Hz) at three current amplitudes (10, 50, or 100 µA) compared with sham stimulation (0 µA, Control) in a fully independent-samples design. After 10 min of stimulation, animals were tested in the EPM, followed by the OFT (with a 5-min interval between tests) (Fig. 1A). In Experiment 2, we investigated the impact of HFS or LFS on fear memory consolidation. Protein-dependent fear memory consolidation occurs within 3 h after fear memory training (Bourtchouladze et al. 1998), and optogenetic studies indicate that the most sensitive period for modulating BLA-dependent memory is immediately after the training protocol (Huff et al. 2013). Since both clinical and pre-clinical evidence demonstrate that very brief BLA stimulation (< 1 min) can modulate the consolidation of relevant stimuli (Bass et al. 2012, 2014; Inman et al. 2018), we performed a 10 minute stimulation session immediately after the fear conditioning protocol with HFS or LFS in independent samples. At 2 and 24 h after fear conditioning, short- and long-term fear memory were tested and compared with baseline (Fig. 1B).

### Animals

Male Wistar rats (2–3 months old, 260–350 g) were provided by the university animal facility (UFSC, Brazil) and housed in four to five per cage under environmentally controlled conditions (12:12-h light-dark cycle, 22 ± 2 °C) with food and water available *ad libitum*. All experimental procedures were approved by the University Animal Care and Use Committee (Protocol 00893, CEUA-UFSC, Brazil).

### Surgical procedure for electrode placement

Monopolar electrodes (NiCr, 0.07 mm^2^, California Fine Wire, USA) were manufactured as described elsewhere (Tan et al. 2010) and unilaterally implanted in the right BLA. Right BLA DBS using a monopolar configuration has been previously shown to successfully reduce hypervigilant behavior in a rat model of PTSD (Langevin et al. 2010; Stidd et al. 2013). Briefly, anesthetized animals (Ketamine 100 mg/kg and Xylazine 10 mg/kg, i.p.) were positioned on the stereotaxic apparatus (Stoelting Co, USA), and an incision was made on the head midline level for skull exposure. A single hole was drilled (Ideal Micro-Drill, USA) over the right BLA (−2.8 mm posterior and − 5.0 mm lateral to the bregma) (Paxinos and Watson 1997), and the electrode cathode was inserted − 8.5 mm ventral to the bregma. A micro-stainless screw was attached to the skull, and the anode was connected to it. Finally, the electrode plug was secured to the skull with dental cement. Animals were allowed to recover for seven days before the experiments.

### Stimulation protocol

Animals were individually placed in a cylindrical cage (40 cm high x 25 cm diameter) and connected to the DBS system (Station5 DBS, InPulse, Brazil). Stimulation intensities (in µA) were chosen based on safety and clinical significance. To determine the safety limits, a pilot study was designed to determine the highest current that could be applied without producing overt nonspecific behavioral signs of pain or discomfort. To this end, incremental current amplitudes (10 µA, 50 µA, 100 µA, 200 µA, and 400 µA) were delivered for up to 1 min, whereas signs of pain/discomfort (i.e., vocalization) were observed at the maximum intensity of 400 µA. Since currents exceeding 150 µA in rodent BLA stimulation have previously been associated with epileptiform after-discharges (Reznikov et al. 2016), we selected stimulation amplitudes below this epileptiform threshold, namely 10, 50, and 100 µA. Considering a fixed pulse width of 90 µs and the electrode area of 0.07 mm^2^ (Brocker and Grill 2013; Kuncel and Grill 2004; Prime et al. 2018), these stimulation currents generated a charge density per pulse of 1.27 µC/cm^2^ (10 µA), 6.36 µC/cm^2^ (50 µA), and 12.73 µC/cm^2^ (100 µA). Importantly, large clinical cohorts report an average clinical charge density of 7.2 µC/cm^2^ for treating Parkinson’s disease and 8.3 µC/cm^2^ for treating essential tremor (Fakhar et al. 2013). In terms of stimulation frequency, HFS tends to result in local tissue inhibition, whereas low-frequency stimulation LFS tends to result in local tissue activation (Benazzouz et al. 1993; Udupa and Chen 2015). Following previously established protocols (Hamani et al. 2010, 2014; Zhang et al. 2024), our study adopted 130 Hz for HFS and 20 Hz for LFS, with a fixed pulse width of 90 µs. Finally, to experimentally simulate the acute clinical protocols used to test for side effects and stimulation tolerability (Greenberg et al. 2010; Schlaepfer et al. 2014), animals were briefly stimulated for 10 min before behavioral testing.

### Elevated plus maze

The elevated plus-maze (EPM) test assesses rodents’ expression of defensive behaviors in response to innate aversive stimuli, such as open and elevated areas (Pellow et al. 1985; Walf and Frye 2007). The EPM has high predictive validity as the rodent’s expression of defensive behaviors is bidirectionally modulated by anxiogenic or anxiolytic treatments (Walf and Frye 2007). The apparatus consists of two opposed closed arms (50 × 10 cm^2^) and two opposed open arms (50 × 10 cm^2^), arranged at a 90° angle, facing a central platform (10 × 10 cm^2^) (Insight Ltda., Brazil). The arms are elevated 40 cm above the floor. Experiments were carried out in dim light (20 lx). Each animal was individually placed on the central platform, facing an enclosed arm, and allowed to explore the apparatus for 5 min (Pellow et al. 1985), while the entire session was video recorded. The recordings were analyzed by an experienced researcher, blinded for the groups, considering the percentages of time and entries in the open arms (a measure of anxiety-like behavior), and the total number of arm entries (a measure of locomotor activity) (Pellow et al. 1985; Rodgers and Dalvi 1997). Ethological variables, such as the frequency of unprotected head-dipping, protected stretched attend posture, rearing, and the total time spent on grooming behavior, were also considered (Carobrez and Bertoglio 2005; Rodgers and Dalvi 1997).

### Open-field test

The open-field test (OFT) was used to measure the animals’ spontaneous locomotor activity (Walsh and Cummins 1976). The apparatus consists of a square arena measuring 1 m^2^ and enclosed by 40-cm-high sidewalls (Insight, Brazil). Each animal was placed in one of the corners, facing the wall, and allowed to explore the arena for 5 min (Suarez and Gallup 1981), while its behavior was video-monitored by an automated system (AnyMaze, Stoelting Co., USA). The OFT center was defined as a virtual square area (50 × 50 cm), while the distance traveled, mean speed, the proportion of time spent in the center, and number of entries in the center area were recorded.

### Fear conditioning

Fear conditioning consists of temporally associating a biologically neutral stimulus (e.g., the context and/or tone) with an unconditioned aversive stimulus (e.g., a foot shock) (Pavlov 1927), whereas the time spent on freezing behavior during re-exposure to the conditioned stimulus is used as an indicator of associative learning and conditioned fear (Izquierdo et al. 2016; LeDoux 2014). The conditioning system consisted of a rectangular chamber (26 × 26 × 30 cm) with a stainless-steel grid floor, connected to a shock generator (EP 107 C, Insight, Brazil). Animals were brought into the conditioning chamber and allowed to habituate for 2 min and 50 s. After this time, a tone was presented for 10 s (1 kHz, 100 dB), co-terminating with a foot shock (0.7 mA, 1 s). Foot shock was delivered a second time after 30 s in the absence of tone, with the entire training session lasting for 4 min. The temporal dynamics of amygdala-dependent memory consolidation have been classically divided into two stages: a short-term memory phase occurring within 3 h after training in which fear memory retrieval is not affected by protein synthesis inhibition, and the protein synthesis-dependent long-term memory that lasts over days or longer (Bourtchouladze et al. 1998; Schafe and LeDoux 2000a). Protocols for studying fear memory consolidation, therefore, have often measured short-term fear memory retrieval 2 h after training and long-term retrieval 24 h after training (Curzon et al. 2009). Accordingly, in our study, animals were re-exposed for 4 min to the same chamber 2 h after fear conditioning to evaluate short-term fear memory. Similarly, 24 h after fear conditioning, animals were re-exposed to the conditioning chamber and tone (10 s, presented once 2 min and 50 s after the animal had been introduced to the chamber) for 4 min to evaluate long-term fear memory. Re-exposures were made in the absence of foot shocks. Fear conditioning and fear memory retrieval sessions were video-recorded and analyzed by an experienced researcher, who was blinded to the groups. Freezing behavior was defined as the absence of movements for a period longer than 1 s, except for respiratory activity. Baseline (non-associative) freezing behavior was computed as the percentage of freezing time relative to the first 2 min and 50 s of exposure to the chamber during fear conditioning (before the first tone presentation). Short-term fear memory was measured as the percentage of freezing behavior relative to the 4 min of re-exposure to the chamber. Long-term fear memory was divided into the percentage of freezing behavior relative to the first 2 min and 50 s of re-exposure to the chamber (context only), and the percentage of freezing behavior relative to the 60 s immediately after tone presentation.

### Statistical analysis

Distribution was assessed using the Shapiro-Wilk test. Data were presented as the mean plus standard error of the mean (SEM). EPM and OFT tests were analyzed using one-way ANOVA (stimulation amplitude as the factor), followed by Tukey’s test for multiple comparisons. Fear memory was analyzed using a two-way repeated-measures ANOVA, with stimulation frequency as the between-subjects factor and freezing behavior across the time points (i.e., baseline, short-term, and long-term memory retention) as repeated measures. Non-normally distributed variables were analyzed using both parametric and non-parametric tests. In cases where significance was not affected, parametric analysis was preferred (Krzywinski and Altman 2014). All analyses were performed using GraphPad Prism 8, considering differences statistically significant when *p ≤* 0.05.

## Results

**Fig. 2:**
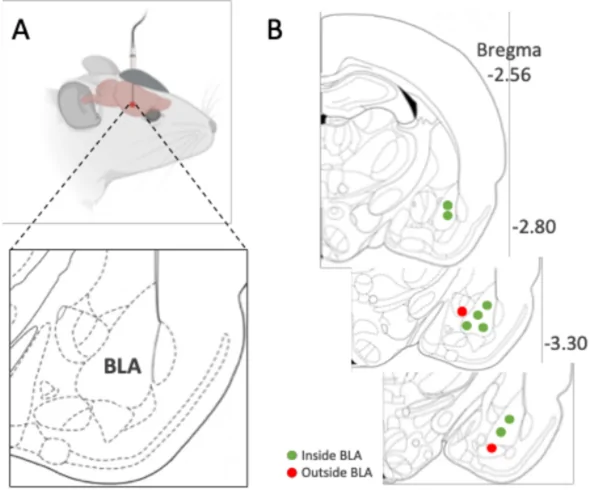
Representation of BLA anatomical location and electrode position. **A** Schematic representation of the electrode montage on the rat’s head (top) targeting the right BLA (bottom). **B** Representative locations of electrode positions across the anteroposterior axis. Green circles represent valid electrode positions while red circles represent electrode misplacements that were excluded from the analysis

One hundred and thirty-five animals were used across experiments. The success rate for correct electrode placement in the BLA was approximately 90%. Figure 2 depicts the representative locations of the most common electrode placement positions across the anteroposterior axis (−2.56 mm to −3.30 mm from Bregma), characterized as inside or outside the targeted brain region. The data from animals with misplaced electrodes were excluded from the analysis.

### Experiment 1: BLA DBS does not affect the expression of anxiety-like behavior

**Fig. 3:**
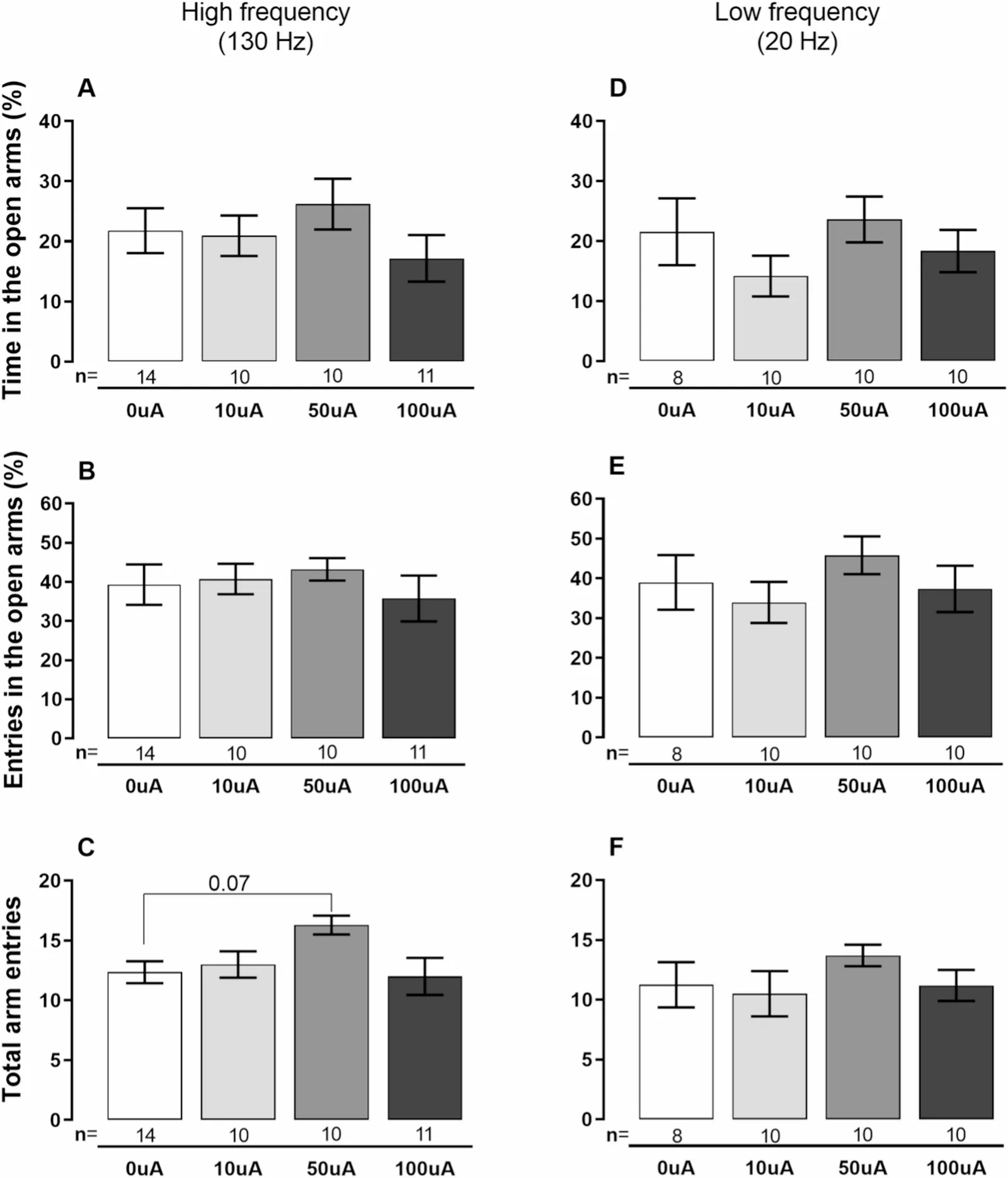
BLA DBS does not affect the expression of anxiety-like behavior in the EPM. Different cohorts of animals were grouped according to stimulation intensity (0 µA as control) and frequency. Percentage of time **A** and entries **B** in the open arms, and total number of arm entries **C** in the EPM after high frequency (130 Hz) DBS. Percentage of time **D** and entries **E** in the open arms, and total number of arm entries **F** in the EPM after low frequency (20 Hz) DBS. Data presented as mean ± S.E.M.; one-way ANOVA followed by Tukey’s post-hoc test

Independent samples were grouped by stimulation intensity (0 [Ctrol], 10, 50, or 100 µA) and frequency (HFS or LFS). Animals were submitted to the EPM and OFT immediately after the 10-minute stimulation period. One-way ANOVA (stimulation amplitude as a factor) indicated no significant differences in the percentages of time (Fig. 3A, F(3, 41) = 0.86, *p* = 0.46) or entries (Fig. 3B, F(3, 41) = 0.39, *p* = 0.76) in the open arms of the EPM for animals stimulated with HFS, regardless of stimulation intensity. Likewise, LFS did not affect the percentages of time (Fig. 3D, F(3, 34) = 1.1, *p* = 0.37) or entries (Fig. 3E, LFS: F (3, 34) = 0.84, *p* = 0.48) in the EPM. These data suggest no significant alterations in the expression of anxiety-like behavior induced by HFS or LFS BLA-DBS across the tested stimulation amplitudes. However, there was a main effect of HFS on the total number of arm entries (Fig. 3C, F(3, 41) = 2.9, *p* = 0.04). Tukey’s post hoc test showed a non-significant increase in the total number of arm entries induced by 50 µA compared with the control group (*p* = 0.07). This effect was not observed for LFS (Fig. 3F, F(3, 34) = 0.89, *p* = 0.45).

**Fig. 4:**
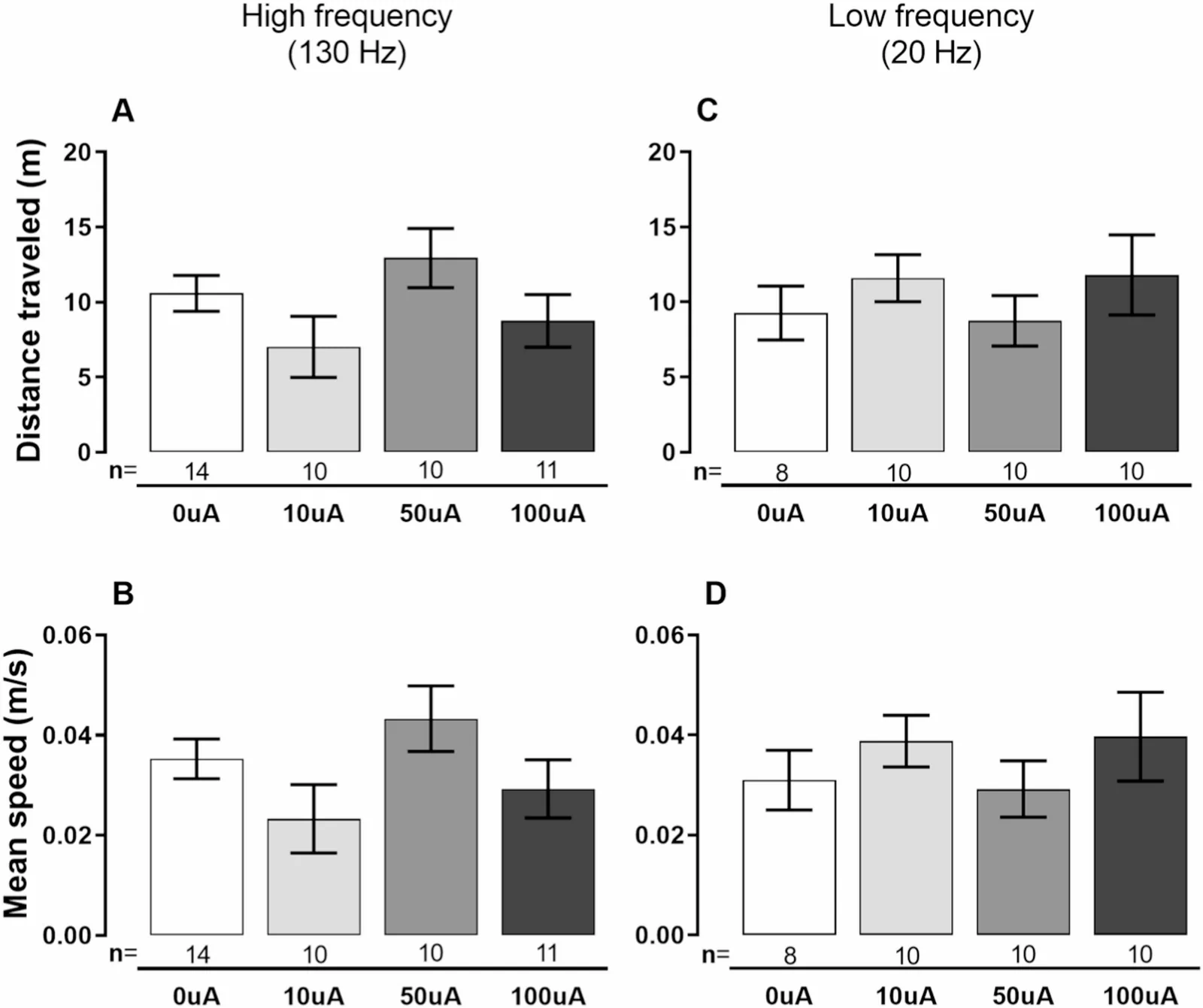
BLA DBS does not affect spontaneous locomotor activity in the OFT. The OFT was performed 5 min after the EPM test. Distance traveled **A** and mean speed **B** after high-frequency (130 Hz) DBS. Distance traveled **C **and mean speed **D** after low-frequency (20 Hz) DBS. Data presented as mean ± S.E.M.; one-way ANOVA

**Fig. 5:**
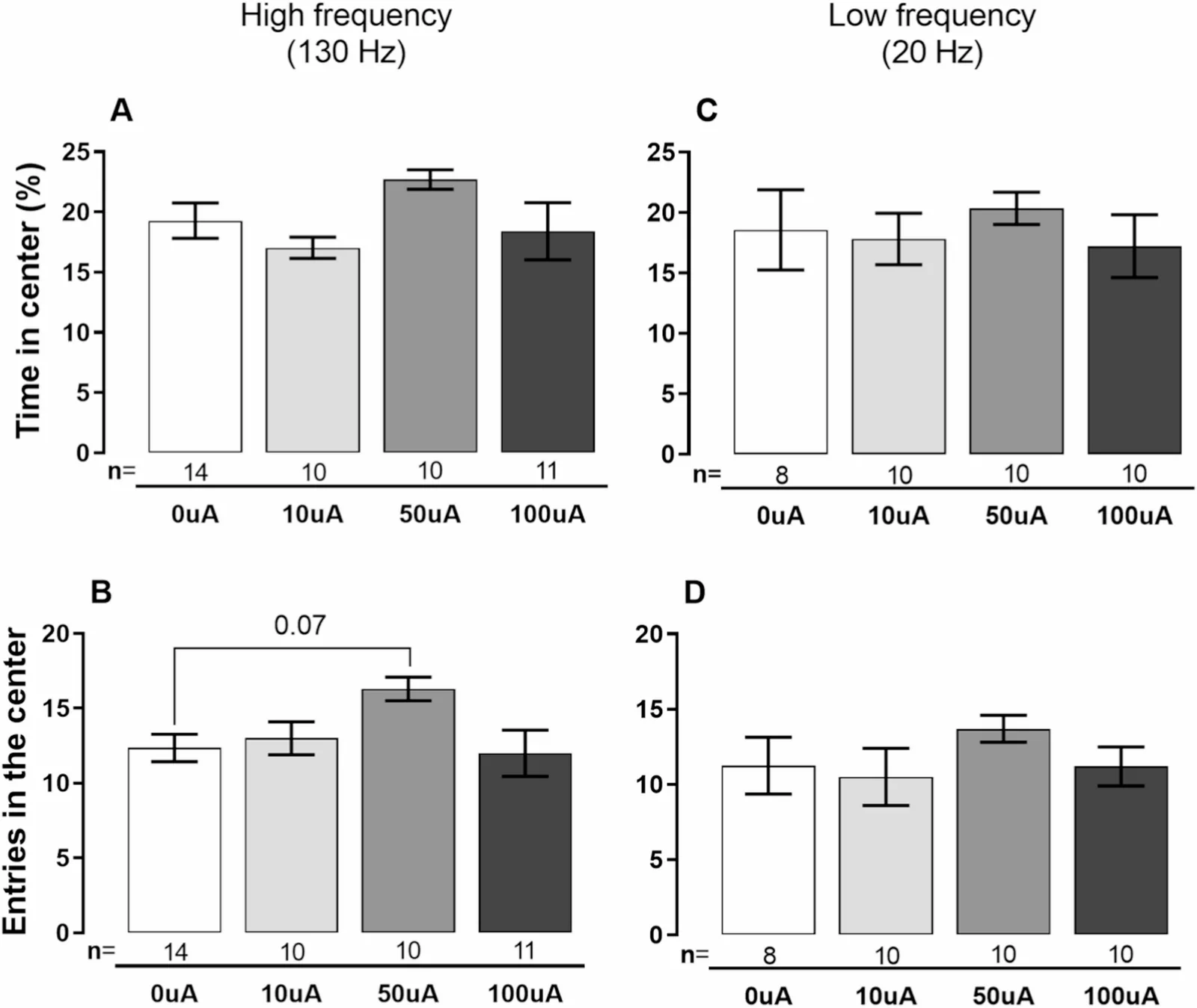
BLA DBS does not affect anxiety-like behavior in the OFT. Time spent in the center of the OFT **A** and number of center entries **B** after high-frequency (130 Hz) DBS. Time spent in the center **C** and center entries **D** after low-frequency (20 Hz) DBS. Data presented as mean ± S.E.M.; one-way ANOVA followed by Tukey’s post-hoc test

Total arm entries in the EPM indicate increased locomotor activity, which can be a confounding factor in interpreting EPM results (Weiss et al. 1998). Therefore, the OFT, which is sensitive to locomotor and exploratory effects (Walsh and Cummins 1976), was performed 5 min after the EPM test to control for such effects. One-way ANOVA (stimulation amplitude as a factor) reported no significant differences for HFS or LFS in terms of distance travelled (Fig. 4A, HFS: F (3, 41) = 2.0, *p* = 0.12; Fig. 4C, LFS: F (3, 34) = 0.63, *p* = 0.60) or mean speed (Fig. 4B, HFS: F (3, 41) = 2.1, *p* = 0.11; Fig. 4D, LFS: F (3, 34) = 0.65, *p* = 0.58), suggesting BLA DBS did not impact locomotor activity. Moreover, the time spent in the center of the arena, which indicates levels of anxiety-like behavior, was not affected by the different stimulation parameters (Fig. 5A, HFS: F (3, 41) = 2.11, *p* = 0.11; Fig. 5C, LFS: F (3, 34) = 0.35, *p* = 0.79), although there was a positive main effect regarding the number of entries in the OFT center for HFS (Fig. 5B, F (3, 41) = 2.9, *p* = 0.05). This effect was mostly driven by a marginal increase in center entries in animals stimulated with 50 µA (Fig. 5B, *p* = 0.07). No changes in center entries were detected after LFS (Fig. 5D, F (3, 34) = 0.9, *p* = 0.46).

### Experiment 2: BLA DBS does not affect short- or long-term fear memory consolidation

**Fig. 6:**
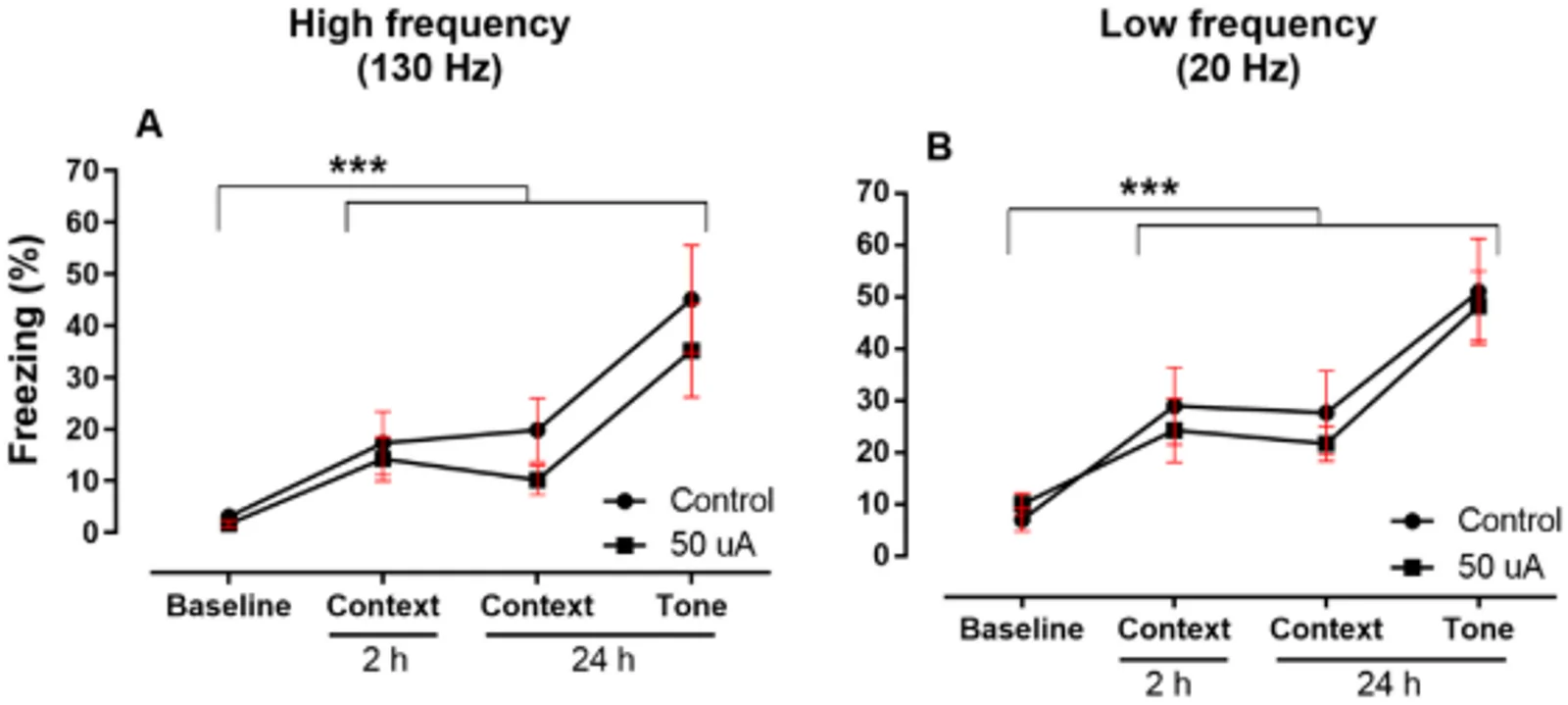
BLA DBS does not affect short- or long-term fear memory consolidation. Ten minutes of 50 µA high-frequency (130 Hz, *n* = 8–10/group) **A** or low-frequency (20 Hz, *n* = 8–10/group) **B** BLA-DBS was applied immediately after fear memory conditioning. The percentage of freezing behavior relative to baseline was used as an indicator of fear memory consolidation. Data presented as mean ± S.E.M. *** *p* < 0.001 for the effect of time (two-way ANOVA with repeated measures)

Although changes in the previously tested behaviors did not reach statistical significance, 50 µA HFS marginally affected the number of arm entries in the EPM and center entries in the OFT. Notably, 50 µA stimulation results in a charge density per pulse (6.36 µC/cm^2^) similar to the average charge densities utilized in clinical applications (~ 7 µC/cm^2^) (Fakhar et al. 2013). Therefore, to investigate the effects of BLA-DBS on short- and long-term fear memory consolidation, 50 µA HFS or LFS were applied for 10 min immediately after fear memory conditioning. Two-way repeated-measures ANOVA indicated a significant effect of time for both stimulation frequencies, as evidenced by increased freezing behavior relative to baseline (Fig. 6A, HFS: F(3, 48) = 21.0, *p* < 0.001; Fig. 6B, LFS: F(3, 48) = 23.0, *p* < 0.001), confirming that the conditioning protocol induced fear learning. In contrast, there was no significant effect for HFS (Fig. 6A, F(1, 16) = 1.1, *p* = 0.31) or LFS (Fig. 6B, F(1, 16) = 0.17, *p* = 0.68) on the expression of freezing behavior compared to controls (0 µA as sham stimulation), suggesting BLA DBS does not affect short-term or long-term fear memory consolidation.

## Discussion

BLA DBS has been proposed as a therapeutic alternative for treatment-resistant PTSD. Nevertheless, the feasibility of BLA DBS may be compromised due to its central relevance in the survival system, modulating responses such as stress, anxiety, and fear. Given the complexity, costs, and risks associated with DBS, experimental investigations are paramount to provide fundamental pre-clinical information, such as safety and tolerability. Here, we aimed to investigate the effects of acute BLA DBS and hypothesized that low-frequency BLA DBS would elicit acute anxiety-like behaviors and enhance fear memory consolidation, whereas HFS would lead to opposite outcomes. Nevertheless, our data suggested that, within the limits of the parameters and protocols utilized in our study, acute unilateral BLA DBS does not affect the expression of anxiety-like behaviors, locomotor activity, or fear memory consolidation. Although they are contrary to our initial hypothesis, these preclinical results may have implications for understanding the tolerability and safety of amygdala DBS.

The experimental investigation of safety and target-associated effects is fundamental for clinical practice. DBS can trigger diverse target-associated side effects, including alterations in smell and taste (Okun et al. 2007), impairment in verbal fluency (Fagundes et al. 2016), and disorders affecting eye movement and speech (Zhang et al. 2010). Even though these effects are often reversed by adjusting the stimulation parameters, such adjustments often hinder the adoption of optimal parameter settings for symptom relief (Larson 2014), as a balance between efficacy and safety must be achieved. Such clinical observations, therefore, suggest the necessity for careful experimental investigation of different stimulation protocols.

High-frequency DBS is commonly regarded as producing an inhibition-like effect in contrast with the activation-like effect induced by LFS (Grill et al. 2004). Indeed, high-frequency DBS (130 Hz) of the amygdala effectively reduces the frequency and intensity of seizures in patients with temporal lobe epilepsy (Boon et al. 2007; Vonck et al. 2002). Moreover, amygdala DBS has been shown to reduce self-injurious behavior in autism spectrum disorder (Sturm et al. 2013), and the severity of PTSD symptoms in war veterans (Koek et al. 2017, 2024; Langevin et al. 2016a, b). On the other hand, LFS DBS (50 Hz) targeting the right amygdala can induce acute fear responses in humans (Lanteaume et al. 2007; Meletti et al. 2006). Likewise, optogenetic activation of the BLA projections to the medial prefrontal cortex (mPFC) in rodents increases the expression of defensive behaviors in the EPM and OFT (Felix-Ortiz et al. 2016). Based on that, we expected low-frequency BLA DBS could elicit anxiogenic responses. However, we found that neither low-frequency nor high-frequency acute unilateral BLA DBS had a lasting effect on anxiety-like behavior, as indicated by similar time spent in the open arms of the EPM compared with controls, regardless of stimulation intensity.

Amygdala activity has also been linked with memory prioritization based on affective salience, such that treatments that increase amygdala excitability after an event also enhance memory consolidation for that event (Paré 2003). Brief BLA stimulation (50 Hz, 20 uA, 1 s) during the rodent novel object recognition test, a non-emotional spatial memory task, increased the long-term memory of the objects that were immediately followed by BLA stimulation compared with those that were not (Bass et al. 2012, 2014). When a similar approach was tested in humans, BLA stimulation (50 Hz, 0.5 mA for 1 s) during the presentation of a random selection of neutral figures increased the recall of such figures compared with those presented without stimulation (Inman et al. 2018). Therefore, BLA DBS could eventually interfere with memory consolidation. Since the amygdala is directly involved in the consolidation of fear memory (LeDoux 2014; Phillips and LeDoux 1992; Schafe and LeDoux 2000b), we investigated whether acute BLA DBS would interfere with this process. Nevertheless, we observed no significant changes in the consolidation of short- or long-term fear memories, regardless of stimulation frequency or intensity. Therefore, brief unilateral high- or low-frequency BLA DBS with a resulting charge density of 6.36 µC/cm^2^ per pulse, applied immediately after an emotionally salient stimulus, does not interfere with the consolidation of such fear-related memory in rodents.

It should be noted that our long-term memory test session re-exposed animals to the auditory stimulus in the same context where fear conditioning took place the day before. This limits the discrimination between contextual and auditory fear conditioning as it conflates freezing attributable to context with freezing attributable to tone. In the context of reconsolidation, Payne, Merza, and Mumby demonstrated that performing the procedures for auditory fear memory reactivation and reconsolidation in the same context can result in second-order conditioning to the context (Payne et al. 2023). Therefore, the protocol we have utilized permits interpreting the short-term fear memory response as contextual (as no tone was presented during the 2 h re-exposure), while long-term fear memory likely represents the compounding effects of context and auditory cue.

Our hypothesis assumed that high-frequency DBS produced an inhibition-like effect while LFS led to an activation-like effect (Grill et al. 2004). This assumption stems from the early local suppression theory, in which high-frequency DBS was suggested to result in local tissue inhibition (Neumann et al. 2023). Other proposed mechanisms account for the long-range effects of DBS, suggesting that high-frequency DBS de-afferents the stimulated nucleus, functionally preventing upstream and downstream communication (Carron et al. 2013). In effect, our understanding of the mechanisms of action of DBS is still evolving, and it is likely that the observed outcomes result from a complex interaction of both local synaptic modulation and long-range network oscillations (Neumann et al. 2023), which also challenges the overly simplistic dichotomy of activation-inhibition previously attributed to stimulation frequency. Indeed, acute high-frequency STN DBS results in antidromic activation of the motor cortex that weans as stimulation continues into a chronic phase (Johnson et al. 2020). Such divergent neurological effects between the acute and chronic phases of DBS underscore the importance of investigating stimulation responses that result not only from chronic DBS.

One limitation of our study is the lack of molecular or histological markers that would demonstrate the extent to which neuronal activity and related networks were affected. For instance, by measuring the expression of *zif268*, a marker of recent neuronal activation, Hamani and colleagues demonstrated that DBS of the rat homologous ventral capsule/ventral striatum (VC/VS) (100 µA, 90 µs, 130 Hz) engaged the activation of cortical and subcortical brain regions despite no significant changes in depression-like behaviors. Nevertheless, we previously demonstrated that pharmacological inhibition of the MEK/ERK signaling pathway within the BLA significantly impairs both the consolidation and the expression of conditioned fear memory, while leaving anxiety-like behavior in the elevated plus maze unchanged (de Carvalho et al. 2021). This indicates that the behavioral paradigms utilized in our study are sensitive to BLA modulation, supporting the understanding that the stimulation protocols utilized in our research (which are comparable to those used in clinical DBS applications) did not reach the threshold to provoke acute behavioral changes.

The lack of support for our hypothesis could stem from methodological aspects. In our protocol, we performed unilateral stimulation (right hemisphere). Therefore, our findings cannot account for the potential effects of bilateral BLA DBS. Nevertheless, clinical evidence suggests that stimulation of the right amygdala induces negative feelings (e.g., fear) (Meletti et al. 2006), while stimulation of the left amygdala induces positive feelings (e.g., pleasure) (Lanteaume et al. 2007). Furthermore, right BLA DBS was effective in reducing the PTSD phenotype in a rodent model (Hashtjini et al. 2018; Langevin et al. 2010; Mokhtari Hashtjini et al. 2018; Stidd et al. 2013). Finally, Hilliard and colleagues demonstrated that right BLA DBS was comparable to bilateral BLA DBS in reducing the rodents’ PTSD phenotype (Hilliard and Foote 2018). Therefore, while unilateral amygdala DBS is sufficient to elicit therapeutic effects, stimulation of the right amygdala could also induce negative-valence effects. In this regard, even though our findings cannot be extrapolated to bilateral BLA DBS, the use of right BLA DBS was a plausible methodological choice given our objectives. Additionally, acute amygdala stimulation (seconds to minutes) is sufficient to elicit stress-related behaviors and increase skin conductance, a biomarker for stress-associated responses (Lanteaume et al. 2007). Similarly, brief (1s) amygdala stimulation is sufficient to alter the efficacy of memory consolidation (Bass et al. 2012, 2014; Inman et al. 2018). This evidence supports the use of acute amygdala DBS as a feasible model for studying immediate stimulation-induced effects.

It is also relevant to note that our protocol examined behavioral effects in response to the stimulation rather than during it, which raises the possibility that the effects of BLA DBS are washed out as soon as the stimulation ceases. In patients with Parkinson’s disease, the DBS effect on tremor and rigidity is almost immediate. When the stimulus is suspended, a sequential pattern of return of parkinsonian signs is observed, with a fast worsening of tremor within minutes, followed by a smoother, slower worsening of bradykinesia and rigidity over half an hour to an hour, and finally a slow and steady worsening of axial signs over 3 to 4 h (Temperli et al. 2003). The complete washout, therefore, requires a few hours after the stimulation is finished (Cooper et al. 2013). Our results suggest that, if acute unilateral BLA DBS eventually affects the behavioral tests we analyzed, the immediate discontinuation of stimulation is sufficient to wash out these effects. Further studies using continuous DBS that can be applied during behavioral task performance are required to exclude behavioral effects that may show faster washout periods.

Finally, our sample was composed exclusively of male rats, limiting the generalization of our findings to females. Although males and females tend to behave similarly in many test paradigms, female behavior in ethologically relevant tests (including the OFT and EPM) can be markedly affected by the estrous cycle (Lovick and Zangrossi 2021). Moreover, although fear memory acquisition and consolidation are generally not influenced by sex (Lovick and Zangrossi 2021), the BLA neurophysiology is responsive to changes in the estrous cycle (Blume et al. 2017). These estrous-related changes could eventually affect the physiological and behavioral response to BLA DBS. Therefore, in exploring sex differences, it would be meaningful for future studies to investigate behavioral changes in response to BLA DBS at the different phases of the estrous cycle.

In summary, the amygdala is a promising DBS target for the treatment of psychiatric and neurological disorders, such as PTSD, ASD, and TLE. However, the potential behavioral and cognitive effects of amygdala DBS remain largely unknown. Here, we demonstrated that acute unilateral BLA DBS is unlikely to produce lasting behavioral or memory-associated changes, considering the parameters and protocols utilized in our study. Nonetheless, important questions remain, such as the effects of bilateral amygdala stimulation and the behavioral changes under continuous stimulation protocols.

## Data Availability

The datasets generated and/or analyzed in the current study are available from the corresponding author upon reasonable request.

## Declarations

### Open practice statement

Data or materials for the experiments are available upon request, and none of the experiments were preregistered.

## References

[CR1] Alonso P, Cuadras D, Gabriëls L, Denys D, Goodman W, Greenberg BD, Jimenez-Ponce F, Kuhn J, Lenartz D, Mallet L, Nuttin B, Real E, Segalas C, Schuurman R, Montcel D, S. T., Menchon JM (2015) Deep brain stimulation for obsessive-compulsive disorder: A meta-analysis of treatment outcome and predictors of response. PLoS ONE 10(7):1–16. 10.1371/journal.pone.0133591 PMC4514753 26208305

[CR3] Bass DI, Partain KN, Manns JR (2012) Event-specific enhancement of memory via brief electrical stimulation to the basolateral complex of the amygdala in rats. Behav Neurosci 126(1):204–208. 10.1037/a0026462 PMC3269500 22141467

[CR2] Bass DI, Nizam ZG, Partain KN, Wang A, Manns JR (2014) Amygdala-mediated enhancement of memory for specific events depends on the hippocampus. Neurobiol Learn Mem 107:37–41. 10.1016/j.nlm.2013.10.020 PMC3888811 24211699

[CR4] Benabid AL, Pollack P, Loveau A, Henry S, de Rougemont J (1987) Combined (Thalamototmy and Stimulation) Stereotactic Surgery of the VM Thalamic Nucleus for Bilateral Parkinson Disease. Stereotact Funct Neurosurg 50(1–6):344–346. 10.1159/000100803 3329873

[CR5] Benazzouz A, Gross C, Féger J, Boraud T, Bioulac B (1993) Reversal of Rigidity and Improvement in Motor Performance by Subthalamic High-frequency Stimulation in MPTP‐treated Monkeys. Eur J Neurosci 5(4):382–389. 10.1111/j.1460-9568.1993.tb00505.x 8261116

[CR6] Blume SR, Freedberg M, Vantrease JE, Chan R, Padival M, Record MJ, De Joseph MR, Urban JH, Rosenkranz JA (2017) Sex- And estrus-dependent differences in rat basolateral amygdala. J Neurosci 37(44):10567–10586. 10.1523/JNEUROSCI.0758-17.2017 PMC5666581 28954870

[CR7] Boon P, Vonck K, de Herdt V, van Dycke A, Goethals M, Goossens L, van Zandijcke M, de Smedt T, Dewaele I, Achten R, Wadman W, Dewaele F, Caemaert J, van Roost D (2007) Deep Brain Stimulation in Patients with Refractory Temporal Lobe Epilepsy. Epilepsia 48(8):1551–1560. 10.1111/j.1528-1167.2007.01005.x 17726798

[CR8] Boraud T, Bezard E, Bioulac B, Gross C (1996) High frequency stimulation of the internal Globus Pallidus (GPi) simultaneously improves parkinsonian symptoms and reduces the firing frequency of GPi neurons in the MPTP-treated monkey. Neurosci Lett 215(1):17–20. 10.1016/S0304-3940(96)12943-8 8880743

[CR9] Bourtchouladze R, Abel T, Berman N, Gordon R, Lapidus K, Kandel ER (1998) Different training procedures recruit either one or two critical periods for contextual memory consolidation, each of which requires protein synthesis and PKA. Learn Memory (Cold Spring Harbor N Y) 5(4–5):365–374. http://www.ncbi.nlm.nih.gov/pubmed/10454361 PMC311273 10454361

[CR10] Brocker DT, Grill WM (2013) Principles of electrical stimulation of neural tissue. In: Handbook of Clinical Neurology (1st ed., Vol. 116, pp. 3–18). Elsevier B.V. 10.1016/B978-0-444-53497-2.00001-2 24112880

[CR11] Carobrez AP, Bertoglio LJ (2005) Ethological and temporal analyses of anxiety-like behavior: The elevated plus-maze model 20 years on. Neurosci Biobehav Rev 29(8):1193–1205. 10.1016/j.neubiorev.2005.04.017 16084592

[CR12] Carron R, Chaillet A, Filipchuk A, Pasillas-Lépine W, Hammond C (2013) Closing the loop of deep brain stimulation. Front Syst Neurosci 7(December):1–18. 10.3389/fnsys.2013.00112 PMC3868949 24391555

[CR13] Cogan SF, Ludwig KA, Welle CG, Takmakov P (2016) Tissue damage thresholds during therapeutic electrical stimulation. J Neural Eng 13(2):021001. 10.1088/1741-2560/13/2/021001 PMC5386002 26792176

[CR14] Cooper SE, McIntyre CC, Fernandez HH, Vitek JL (2013) Association of Deep Brain Stimulation Washout Effects With Parkinson Disease Duration. JAMA Neurol 70(1):95. 10.1001/jamaneurol.2013.581 PMC5148628 23070397

[CR15] Coubes P, Roubertie A, Vayssiere N, Hemm S, Echenne B (2000) Treatment of DYT1-generalised dystonia by stimulation of the internal globus pallidus. Lancet 355(9222):2220–2221. 10.1016/S0140-6736(00)02410-7 10881900

[CR16] Curzon P, Rustay NR, Browman KE (2009) Cued and Contextual Fear Conditioning for Rodents. Methods of Behavior Analysis in Neuroscience. CRC Press/Taylor & Francis. http://www.ncbi.nlm.nih.gov/pubmed/21204331 21204331

[CR17] de Carvalho CR, Lopes MW, Constantino LC, Hoeller AA, de Melo HM, Guarnieri R, Linhares MN, Bortolotto ZA, Prediger RD, Latini A, Lin K, Licinio J, Leal RB, Walz R (2021) The ERK phosphorylation levels in the amygdala predict anxiety symptoms in humans and MEK/ERK inhibition dissociates innate and learned defensive behaviors in rats. Mol Psychiatry 26(12):7257–7269. 10.1038/s41380-021-01203-0 34316004

[CR18] Duvarci S, Pare D (2014) Amygdala Microcircuits Controlling Learned Fear. Neuron 82(5):966–980. 10.1016/j.neuron.2014.04.042 PMC4103014 24908482

[CR19] Fagundes VDC, Rieder CRM, Cruz AN, Da, Beber BC, Portuguez MW (2016) Deep brain stimulation frequency of the subthalamic nucleus affects phonemic and action fluency in Parkinson’s Disease. Parkinson’s Disease. 10.1155/2016/6760243 PMC5165157 28050309

[CR20] Fakhar K, Hastings E, Butson CR, Foote KD, Zeilman P, Okun MS (2013) Management of Deep Brain Stimulator Battery Failure: Battery Estimators, Charge Density, and Importance of Clinical Symptoms. PLoS ONE 8(3):e58665. 10.1371/journal.pone.0058665 PMC3594176 23536810

[CR21] Famm K, Litt B, Tracey KJ, Boyden ES, Slaoui M (2013) A jump-start for electroceuticals. Nature 496(7444):159–161. 10.1038/496159a PMC4179459 23579662

[CR22] Felix-Ortiz AC, Burgos-Robles A, Bhagat ND, Leppla CA, Tye KM (2016) Bidirectional modulation of anxiety-related and social behaviors by amygdala projections to the medial prefrontal cortex. Neuroscience 321:197–209. 10.1016/j.neuroscience.2015.07.041 PMC4721937 26204817

[CR23] Fisher R, Salanova V, Witt T, Worth R, Henry T, Gross R, Oommen K, Osorio I, Nazzaro J, Labar D, Kaplitt M, Sperling M, Sandok E, Neal J, Handforth A, Stern J, DeSalles A, Chung S, Shetter A, Young C (2010) Electrical stimulation of the anterior nucleus of thalamus for treatment of refractory epilepsy. Epilepsia 51(5):899–908. 10.1111/j.1528-1167.2010.02536.x 20331461

[CR24] Formolo DA, Gaspar JM, Melo HM, Eichwald T, Zepeda RJ, Latini A, Okun MS, Walz R (2019) Deep Brain Stimulation for Obesity: A Review and Future Directions. Front NeuroSci. 10.3389/fnins.2019.00323 PMC6482165 31057350

[CR25] Fox AS, Shackman AJ (2024) An Honest Reckoning With the Amygdala and Mental Illness. Am J Psychiatry 181(12):1059–1075. 10.1176/appi.ajp.20240941 PMC11611071 39616453

[CR26] Gotman J, Levtova V (1996) Amygdala-hippocampus relationships in temporal lobe seizures: a phase-coherence study. Epilepsy Res 25(1):51–57. 10.1016/0920-1211(96)00021-6 8886661

[CR27] Greenberg BD, Gabriels LA, Malone DA, Rezai AR, Friehs GM, Okun MS, Shapira NA, Foote KD, Cosyns PR, Kubu CS, Malloy PF, Salloway SP, Giftakis JE, Rise MT, Machado AG, Baker KB, Stypulkowski PH, Goodman WK, Rasmussen SA, Nuttin BJ (2010) Deep brain stimulation of the ventral internal capsule/ventral striatum for obsessive-compulsive disorder: worldwide experience. Mol Psychiatry 15(1):64–79. 10.1038/mp.2008.55 PMC3790898 18490925

[CR28] Grill WM, Snyder AN, Miocinovic S (2004) Deep brain stimulation creates an informational lesion of the stimulated nucleus. NeuroReport 15(7):1137–1140. 10.1097/00001756-200405190-00011 15129161

[CR31] Hamani C, Temel Y (2012) Deep Brain Stimulation for Psychiatric Disease: Contributions and Validity of Animal Models. Sci Transl Med 4(142) 142rv8–142rv8. 10.1126/scitranslmed.3003722 22786683

[CR30] Hamani C, Diwan M, Isabella S, Lozano AM, Nobrega JN (2010) Effects of different stimulation parameters on the antidepressant-like response of medial prefrontal cortex deep brain stimulation in rats. J Psychiatr Res 44(11):683–687. 10.1016/j.jpsychires.2009.12.010 20096858

[CR29] Hamani C, Amorim BO, Wheeler AL, Diwan M, Driesslein K, Covolan L, Butson CR, Nobrega JN (2014) Deep brain stimulation in rats: Different targets induce similar antidepressant-like effects but influence different circuits. Neurobiol Dis 71:205–214. 10.1016/j.nbd.2014.08.007 PMC5756089 25131446

[CR32] Hariz MI, Hariz G-M (2013) Therapeutic stimulation versus ablation (pp. 63–71). 10.1016/B978-0-444-53497-2.00006-1 24112885

[CR33] Hashtjini MM, Jahromi GP, Sadr SS, Meftahi GH, Hatef B, Javidnazar D (2018) Deep brain stimulation in a rat model of post-traumatic stress disorder modifies forebrain neuronal activity and serum corticosterone. Iran J Basic Med Sci 21(4):370–375. 10.22038/IJBMS.2018.27482.6705 PMC5960752 29796219

[CR34] Hennessey T, Andari E, Rainnie DG (2018) RDoC-based categorization of amygdala functions and its implications in autism. Neurosci Biobehavioral Reviews 90:115–129. 10.1016/j.neubiorev.2018.04.007 PMC6250055 29660417

[CR35] Hilliard JD, Foote KD (2018) Bilateral amygdala stimulation reduces avoidance behavior in a predator scent posttraumatic stress disorder model. NeuroSurg Focus 45(2):E17. 10.3171/2018.5.FOCUS18266 30064318

[CR36] Huff ML, Miller RL, Deisseroth K, Moorman DE, LaLumiere RT (2013) Posttraining optogenetic manipulations of basolateral amygdala activity modulate consolidation of inhibitory avoidance memory in rats. Proc Natl Acad Sci 110(9):3597–3602. 10.1073/pnas.1219593110 PMC3587230 23401523

[CR37] Iacono RP, Lonser RR, Mandybur G, Yamada S (1995) Stimulation of the globus pallidus in Parkinson’s disease. Br J Neurosurg 9(4):505–510 10.1080/02688699550041142 7576277

[CR38] Inman CS, Manns JR, Bijanki KR, Bass DI, Hamann S, Drane DL, Fasano RE, Kovach CK, Gross RE, Willie JT (2018) Direct electrical stimulation of the amygdala enhances declarative memory in humans. Proc Natl Acad Sci 115(1):98–103. 10.1073/pnas.1714058114 PMC5776809 29255054

[CR40] Izquierdo I, Medina JH, Bianchin M, Walz R, Zanatta MS, Silva D, Bueno RC, Silva M, Ruschel E, Paczko AC N (1993) Memory processing by the limbic system: role of specific neurotransmitter systems. Behav Brain Res 58(1–2):91–98 10.1016/0166-4328(93)90093-6 7907882

[CR39] Izquierdo I, Furini CRG, Myskiw JC (2016) Fear Memory. Physiol Rev 96(2):695–750. 10.1152/physrev.00018.2015 26983799

[CR41] Janak PH, Tye KM (2015) From circuits to behaviour in the amygdala. Nature 517(7534):284–292. 10.1038/nature14188 PMC4565157 25592533

[CR42] Johnson LA, Wang J, Nebeck SD, Zhang J, Johnson MD, Vitek JL (2020) Direct Activation of Primary Motor Cortex during Subthalamic But Not Pallidal Deep Brain Stimulation. J Neurosci 40(10):2166–2177. 10.1523/JNEUROSCI.2480-19.2020 PMC7055133 32019827

[CR44] Koek RJ, Langevin J, Krahl SE, Kosoyan HJ, Schwartz HN, Chen JW, Melrose R, Mandelkern MJ, Sultzer D (2014) Deep brain stimulation of the basolateral amygdala for treatment-refractory combat post-traumatic stress disorder (PTSD): study protocol for a pilot randomized controlled trial with blinded, staggered onset of stimulation. Trials 15(1):356. 10.1186/1745-6215-15-356 PMC4168122 25208824

[CR45] Koek RJ, Langevin J-P, Krahl SE, Chen JWY, Kulick AD, Schwartz HN, Mandelkern MA, Sultzer DL (2017) Amygdala DBS for PTSD: 2 years of observations on the first case. Brain Stimul 10(2):369. 10.1016/j.brs.2017.01.086

[CR43] Koek RJ, Avecillas-Chasin J, Krahl SE, Chen JW, Sultzer DL, Kulick AD, Mandelkern MA, Malpetti M, Gordon HL, Landry HN, Einstein EH, Langevin JP (2024) Deep brain stimulation of the amygdala for treatment-resistant combat post-traumatic stress disorder: Long-term results. J Psychiatr Res 175:131–139. 10.1016/j.jpsychires.2024.05.008 PMC11419692 38733927

[CR46] Krzywinski M, Altman N (2014) Nonparametric tests. Nat Methods 11(5):467–468. 10.1038/nmeth.2937 24820360

[CR47] Kuncel AM, Grill WM (2004) Selection of stimulus parameters for deep brain stimulation. Clin Neurophysiol 115(11):2431–2441. 10.1016/j.clinph.2004.05.031 15465430

[CR48] Lai G, Langevin JP, Koek RJ, Krahl SE, Bari AA, Chen JWY (2020) Acute Effects and the Dreamy State Evoked by Deep Brain Electrical Stimulation of the Amygdala: Associations of the Amygdala in Human Dreaming, Consciousness, Emotions, and Creativity. Front Hum Neurosci. 10.3389/fnhum.2020.00061 PMC7052301 32158384

[CR50] Langevin J-P, De Salles AAF, Kosoyan HP, Krahl SE (2010) Deep brain stimulation of the amygdala alleviates post-traumatic stress disorder symptoms in a rat model. J Psychiatr Res 44(16):1241–1245. 10.1016/j.jpsychires.2010.04.022 20537659

[CR49] Langevin J-P, Chen J, Koek R, Sultzer D, Mandelkern M, Schwartz H, Krahl S (2016a) Deep Brain Stimulation of the Basolateral Amygdala: Targeting Technique and Electrodiagnostic Findings. Brain Sci 6(3):28. 10.3390/brainsci6030028 PMC5039457 27517963

[CR51] Langevin J-P, Koek RJ, Schwartz HN, Chen JWY, Sultzer DL, Mandelkern MA, Kulick AD, Krahl SE (2016b) Deep Brain Stimulation of the Basolateral Amygdala for Treatment-Refractory Posttraumatic Stress Disorder. Biol Psychiatry 79(10):e82–e84. 10.1016/j.biopsych.2015.09.003 26475671

[CR52] Lanteaume L, Khalfa S, Regis J, Marquis P, Chauvel P, Bartolomei F (2007) Emotion Induction After Direct Intracerebral Stimulations of Human Amygdala. Cereb Cortex 17(6):1307–1313. 10.1093/cercor/bhl041 16880223

[CR53] Larson PS (2014) Deep Brain Stimulation for Movement Disorders. In: Neurotherapeutics, Vol. 11, Number 3. Springer New York LLC, pp. 465–474 10.1007/s13311-014-0274-1 PMC4121453 24833244

[CR54] Leal RB, Lopes MW, Formolo DA, de Carvalho CR, Hoeller AA, Latini A, Sousa DS, Wolf P, Prediger RD, Bortolotto ZA, Linhares MN, Lin K, Walz R (2018) Amygdala levels of the GluA1 subunit of glutamate receptors and its phosphorylation state at serine 845 in the anterior hippocampus are biomarkers of ictal fear but not anxiety. Mol Psychiatry. 10.1038/s41380-018-0084-7 29880883

[CR55] LeDoux J (2012) Rethinking the Emotional Brain. Neuron 73(4):653–676. 10.1016/j.neuron.2012.02.004 PMC3625946 22365542

[CR56] LeDoux JE (2014) Coming to terms with fear. Proc Natl Acad Sci USA 111(8):2871–2878. 10.1073/pnas.1400335111 PMC3939902 24501122

[CR57] Lovick TA, Zangrossi H (2021) Effect of Estrous Cycle on Behavior of Females in Rodent Tests of Anxiety. In: Frontiers in Psychiatry, (Vol. 12). Frontiers Media S.A. 10.3389/fpsyt.2021.711065 PMC8438218 34531768

[CR58] Luigjes J, De Kwaasteniet BP, De Koning PP, Oudijn MS, Van Den Munckhof P, Schuurman PR, Denys D (2013) Surgery for psychiatric disorders. World Neurosurg 80(3–4):S31e17–S31e28. 10.1016/j.wneu.2012.03.009 22465369

[CR59] Meletti S, Tassi L, Mai R, Fini N, Tassinari CA, Russo G, Lo (2006) Emotions Induced by Intracerebral Electrical Stimulation of the Temporal Lobe. Epilepsia 47(s5):47–51. 10.1111/j.1528-1167.2006.00877.x 17239106

[CR60] Meoni S, Fraix V, Castrioto A, Benabid AL, Seigneuret E, Vercueil L, Pollak P, Krack P, Chevrier E, Chabardes S, Moro E (2017) Pallidal deep brain stimulation for dystonia: a long term study. J Neurol Neurosurg Psychiatry 88(11):960–967. 10.1136/jnnp-2016-315504 28972096

[CR61] Miocinovic S, Somayajula S, Chitnis S, Vitek JL (2013) History, applications, and mechanisms of deep brain stimulation. JAMA Neurol 70(2):163–171. 10.1001/2013.jamaneurol.45 23407652

[CR62] Mokhtari Hashtjini M, Pirzad Jahromi G, Meftahi GH, Esmaeili D, Javidnazar D (2018) Aqueous extract of saffron administration along with amygdala deep brain stimulation promoted alleviation of symptoms in post-traumatic stress disorder (PTSD) in rats. Avicenna J Phytomedicine 8(4):358–369. http://www.ncbi.nlm.nih.gov/pubmed/30377594 PMC6204151 30377594

[CR63] Neumann W-J, Steiner LA, Milosevic L (2023) Neurophysiological mechanisms of deep brain stimulation across spatiotemporal resolutions. Brain 146(11):4456–4468. 10.1093/brain/awad239 PMC10629774 37450573

[CR64] Odekerken VJJ, van Laar T, Staal MJ, Mosch A, Hoffmann CFE, Nijssen PCG, Beute GN, van Vugt JPP, Lenders MWPM, Contarino MF, Mink MSJ, Bour LJ, van den Munckhof P, Schmand BA, de Haan RJ, Schuurman PR, de Bie RMA (2013) Subthalamic nucleus versus globus pallidus bilateral deep brain stimulation for advanced Parkinson’s disease (NSTAPS study): A randomised controlled trial. Lancet Neurol 12(1):37–44. 10.1016/S1474-4422(12)70264-8 23168021

[CR65] Okun MS, Mann G, Foote KD, Shapira NA, Bowers D, Springer U, Knight W, Martin P, Goodman WK (2007) Deep brain stimulation in the internal capsule and nucleus accumbens region: Responses observed during active and sham programming. J Neurol Neurosurg Psychiatry 78(3):310–314. 10.1136/jnnp.2006.095315 PMC2117652 17012341

[CR66] Paré D (2003) Role of the basolateral amygdala in memory consolidation. Prog Neurobiol 70(5):409–420. 10.1016/S0301-0082(03)00104-7 14511699

[CR67] Pavlov IP (1927) Conditioned reflexes: An investigation of the physiological activity of the cerebral cortex. Annals Neurosciences 17(3):136–141. 10.5214/ans.0972-7531.1017309 PMC4116985 25205891

[CR68] Paxinos G, Watson C (1997) The rat brain in stereotaxic coordinates, 3rd edn. Academic 10.1016/0165-0270(80)90021-7 6110810

[CR69] Payne JW, Merza D, Mumby DG (2023) The standard reconsolidation protocol for auditory fear-conditioning does not account for fear to the test context. PLoS ONE 18(6):e0287193. 10.1371/journal.pone.0287193 PMC10286967 37347748

[CR70] Pellow S, Chopin P, File SE, Briley M (1985) Validation of open: closed arm entries in an elevated plus-maze as a measure of anxiety in the rat. J Neurosci Methods 14(3):149–167. 10.1016/0165-0270(85)90031-7 2864480

[CR71] Pena C, Bowsher K, Costello A, De Luca R, Doll S, Li K, Schroeder M, Stevens T (2007) An Overview of FDA Medical Device Regulation as it Relates to Deep Brain Stimulation Devices. IEEE Trans Neural Syst Rehabil Eng 15(3):421–424. 10.1109/TNSRE.2007.903973 17894274

[CR72] Phillips RG, LeDoux JE (1992) Differential contribution of amygdala and hippocampus to cued and contextual fear conditioning. Behav Neurosci 106(2):274–285. 10.1037/0735-7044.106.2.274 1590953

[CR73] Prime D, Rowlands D, O’Keefe S, Dionisio S (2018) Considerations in performing and analyzing the responses of cortico-cortical evoked potentials in stereo-EEG. Epilepsia 59(1):16–26. 10.1111/epi.13939 29143307

[CR74] Ranck JB (1975) Which elements are excited in electrical stimulation of mammalian central nervous system: A review. Brain Res 98(3):417–440. 10.1016/0006-8993(75)90364-9 1102064

[CR75] Reznikov R, Binko M, Nobrega JN, Hamani C (2016) Deep Brain Stimulation in Animal Models of Fear, Anxiety, and Posttraumatic Stress Disorder. Neuropsychopharmacology 41(12):2810–2817. 10.1038/npp.2016.34 PMC5061888 26932819

[CR76] Rodgers RJ, Dalvi A (1997) Anxiety, defence and the elevated plus-maze. Neurosci Biobehav Rev 21(6):801–810. 10.1016/S0149-7634(96)00058-9 9415905

[CR77] Ross DA, Arbuckle MR, Travis MJ, Dwyer JB, van Schalkwyk GI, Ressler KJ (2017) An Integrated Neuroscience Perspective on Formulation and Treatment Planning for Posttraumatic Stress Disorder. JAMA Psychiatry 74(4):407. 10.1001/jamapsychiatry.2016.3325 PMC5504531 28273291

[CR78] Schafe GE, LeDoux JE (2000a) Memory Consolidation of Auditory Pavlovian Fear Conditioning Requires Protein Synthesis and Protein Kinase A in the Amygdala. J Neurosci 20(18):RC96–RC96. 10.1523/JNEUROSCI.20-18-j0003.2000 PMC6772816 10974093

[CR79] Schafe GE, LeDoux JE (2000b) Memory consolidation of auditory pavlovian fear conditioning requires protein synthesis and protein kinase A in the amygdala. J Neuroscience: Official J Soc Neurosci 20(18):RC96. 10.1523/JNEUROSCI.20-18-j0003.2000 PMC6772816 10974093

[CR80] Schlaepfer TE, Bewernick BH, Kayser S, Hurlemann R, Coenen VA (2014) Deep Brain Stimulation of the Human Reward System for Major Depression—Rationale, Outcomes and Outlook. Neuropsychopharmacology 39(6):1303–1314. 10.1038/npp.2014.28 PMC3988559 24513970

[CR81] Schruers K, Baldi S, van den Heuvel T, Goossens L, Luyten L, Leentjens AFG, Ackermans L, Temel Y, Viechtbauer W (2019) The effects of deep-brain non-stimulation in severe obsessive-compulsive disorder: an individual patient data meta-analysis. In: Translational Psychiatry, (Vol. 9, Number 1). Nature Publishing Group 10.1038/s41398-019-0522-6 PMC6683131 31383848

[CR82] Shapira NA, Okun MS, Wint D, Foote KD, Byars JA, Bowers D, Springer US, Lang PJ, Greenberg BD, Haber SN, Goodman WK (2006) Panic and fear induced by deep brain stimulation. J Neurol Neurosurg Psychiatry 77(3):410–412. 10.1136/jnnp.2005.069906 PMC2077710 16484657

[CR83] Shepherd RK, Villalobos J, Burns O, Nayagam DAX (2018) The development of neural stimulators: a review of preclinical safety and efficacy studies. J Neural Eng 15(4):041004. 10.1088/1741-2552/aac43c PMC6049833 29756600

[CR84] Stidd DA, Vogelsang K, Krahl SE, Langevin J-P, Fellous J-M (2013) Amygdala Deep Brain Stimulation Is Superior to Paroxetine Treatment in a Rat Model of Posttraumatic Stress Disorder. Brain Stimul 6(6):837–844. 10.1016/j.brs.2013.05.008 23835167

[CR85] Sturm V, Fricke O, Bührle CP, Lenartz D, Maarouf M, Treuer H, Mai JK, Lehmkuhl G (2013) DBS in the basolateral amygdala improves symptoms of autism and related self-injurious behavior: a case report and hypothesis on the pathogenesis of the disorder. Front Hum Neurosci. 10.3389/fnhum.2012.00341 PMC3549527 23346052

[CR86] Suarez SD, Gallup GG (1981) An ethological analysis of open-field behavior in rats and mice. Learn Motiv 12(3):342–363. 10.1016/0023-9690(81)90013-8

[CR87] Sui L, Huang S, Peng B, Ren J, Tian F, Wang Y (2014) Deep brain stimulation of the amygdala alleviates fear conditioning-induced alterations in synaptic plasticity in the cortical-amygdala pathway and fear memory. J Neural Transm 121(7):773–782. 10.1007/s00702-014-1183-5 24610492

[CR88] Tan SK, Vlamings R, Lim L, Sesia T, Janssen ML, Steinbusch HW, Visser-Vandewalle V, Temel Y (2010) Experimental Deep Brain Stimulation in Animal Models. Neurosurgery 67(4):1073–1080. 10.1227/NEU.0b013e3181ee3580 20881571

[CR89] Temperli P, Ghika J, Villemure J-G, Burkhard PR, Bogousslavsky J, Vingerhoets FJG (2003) How do parkinsonian signs return after discontinuation of subthalamic DBS? Neurology 60(1):78–81. 10.1212/WNL.60.1.78 12525722

[CR90] Udupa K, Chen R (2015) The mechanisms of action of deep brain stimulation and ideas for the future development. Prog Neurobiol 133:27–49. 10.1016/j.pneurobio.2015.08.001 26296674

[CR91] Volkmann J, Albanese A, Kulisevsky J, Tornqvist AL, Houeto JL, Pidoux B, Bonnet AM, Mendes A, Benabid AL, Fraix V, van Blercom N, Xie J, Obeso J, Rodriguez-Oroz MC, Guridi J, Schnitzler A, Timmermann L, Gironell AA, Molet J, Agid Y (2009) Long-term effects of pallidal or subthalamic deep brain stimulation on quality of life in Parkinson’s disease. Mov Disord 24(8):1154–1161. 10.1002/mds.22496 19412954

[CR92] Vonck K, Boon P, Achten E, de Reuck J, Caemaert J (2002) Long-term amygdalohippocampal stimulation for refractory temporal lobe epilepsy. Ann Neurol 52(5):556–565. 10.1002/ana.10323 12402252

[CR93] Walf AA, Frye CA (2007) The use of the elevated plus maze as an assay of anxiety-related behavior in rodents. Nat Protoc 2(2):322–328. 10.1038/nprot.2007.44 PMC3623971 17406592

[CR94] Walsh RN, Cummins RA (1976) The Open-Field Test: a critical review. Psychol Bull 83(3):482–504. 10.1037/0033-2909.83.3.482 17582919

[CR95] Walz R, Roesler R, Quevedo J, Sant’Anna MK, Madruga M, Rodrigues C, Gottfried C, Medina JH, Izquierdo I (2000) Time-dependent impairment of inhibitory avoidance retention in rats by posttraining infusion of a mitogen-activated protein kinase kinase inhibitor into cortical and limbic structures. Neurobiol Learn Mem 73(1):11–20. 10.1006/nlme.1999.3913 10686120

[CR96] Weiss SM, Wadsworth G, Fletcher A, Dourish CT (1998) Utility of ethological analysis to overcome locomotor confounds in elevated maze models of anxiety. Neurosci Biobehav Rev 23(2):265–271. 10.1016/S0149-7634(98)00027-X 9884119

[CR97] Wu Y-C, Liao Y-S, Yeh W-H, Liang S-F, Shaw F-Z (2021) Directions of Deep Brain Stimulation for Epilepsy and Parkinson’s Disease. Front NeuroSci. 10.3389/fnins.2021.680938 PMC8236576 34194295

[CR98] Zhang K, Bhatia S, Oh MY, Cohen D, Angle C, Whiting D (2010) Long-term results of thalamic deep brain stimulation for essential tremor. J Neurosurg 112(6):1271–1276. 10.3171/2009.10.JNS09371 19911883

[CR99] Zhang KK, Matin R, Gorodetsky C, Ibrahim GM, Gouveia FV (2024) Systematic review of rodent studies of deep brain stimulation for the treatment of neurological, developmental and neuropsychiatric disorders. Translational Psychiatry 14(1):186. 10.1038/s41398-023-02727-5 PMC11009311 38605027

